# Response to Madans *et al*. Comments on Sabariego *et al*. Measuring Disability: Comparing the Impact of Two Data Collection Approaches on Disability Rates. *Int. J. Environ. Res. Public Health*, 2015, *12*, 10329–10351

**DOI:** 10.3390/ijerph13010066

**Published:** 2015-12-22

**Authors:** Carla Sabariego, Cornelia Oberhauser, Aleksandra Posarac, Jerome Bickenbach, Nenad Kostanjsek, Somnath Chatterji, Alana Officer, Michaela Coenen, Lay Chhan, Alarcos Cieza

**Affiliations:** 1 Department of Medical Informatics, Biometry and Epidemiology—IBE, Chair for Public Health and Health Services Research, Research Unit for Biopsychosocial Health, Ludwig-Maximilians-University (LMU), Munich 81377, Germany; cornelia.oberhauser@med.lmu.de (C.O.); michaela.coenen@med.lmu.de (M.C.); 2 Social Protection and Labor, Human Development Network, The World Bank, Washington, DC 20433, USA; aposarac@worldbank.org; 3 Swiss Paraplegic Research, Nottwil 6207, Switzerland; jerome.bickenbach@paraplegie.ch; 4 Data Standards and Informatics (DSI), Department of Information, Evidence and Research (IER), World Health Organization, Geneva 1211, Switzerland; kostanjsekn@who.int; 5 Department of Health Statistics and Information Systems, World Health Organization, Geneva 1211, Switzerland; chatterjis@who.int; 6 Ageing and Life Course Unit, World Health Organization, Geneva 1211, Switzerland; officera@who.int; 7 National Institute of Statistics, Phnom Penh 12301, Cambodia; lay.chhan@gmail.com; 8 Blindness and Deafness Prevention, Disability and Rehabilitation (BDD), World Health Organization, Geneva 1211, Switzerland; acieza@who.int

We greatly appreciate and wish to thank Madans, Mont and Loeb for the issues they raise in their Comment [1] on our paper “Measuring Disability: Using the WHO Model Disability Survey to Address the Impact of Screeners on Disability Rates” [2].

As far as we can determine, however, their comment does not deal with the rationale, methodology or objectives of our paper. Instead they respond to concerns we raise about the use of screeners focusing on the—in our manuscript exemplary—use of the short set of questions developed by the Washington Group on Disability Statistics (WG), hereafter WG-6, as a screener tool. Moreover, the issues raised by Madans, Mont and Loeb focus on a direct comparison between the WG-6 and the tool we have applied, namely the WHO Model Disability Survey (MDS), a survey developed as a collaborative effort of WHO and the World Bank (WB) in response to calls for improved disability data collection.

We are pleased to see that Madans, Mont and Loeb have made two points clear about the intended use of the WG short set of questions that we were somewhat cautious in ascribing to the WG enterprise: The WG-6 ask about the difficulties people have in six domains—seeing, hearing, walking or climbing steps, remembering or concentrating, washing all over or dressing and communicating—directly caused by their state of health. In their comment, the authors state that these questions are intended to function as screeners in disability surveys in order to “*maximize the chances that the population of interest will be captured*”. The second assertion is that these questions are intended to screen for the population “*which, due to difficulty functioning in core domains, is at risk of restricted participation in a non-accommodating environment*”. The population identified with the WG-6 is not necessarily the population of people who actually experience disability in the sense of restricted participation; it is the population who is vulnerable to restricted participation. Thus, they claim to give a prevalence of disability but, in fact, they admit that they give a prevalence of people who are at risk of having a disability.

In our manuscript, we analytically demonstrate the (negative) impact of using screeners to identify a population experiencing disability. We fully agree with Madans, Mont and Loeb though that if a screener is used, then a key issue is “*whether the screener identifies the correct population*”. Indeed, a “screener” should have high sensitivity in identifying the population of interest while not necessarily being highly specific. To account for that, every “screener” tool must be validated against a “gold standard” instrument and have sound and reliable psychometric properties. For instance, the validity of the Kessler Screening Scale for Psychological Distress (K6), a widely implemented brief screener tool for serious mental illness, has been examined in several studies against standardized clinical ratings used as the gold standards, such as the Structured Clinical Interviews for DSM-IV, and yet it is clear that for every new setting this tool will need to be validated to serve its purpose of screening the right populations [3,4]. Neither sensitivity and specificity nor psychometric properties of the WG-6 screener have been published so far. Madans, Mont and Loeb make much of the claim that the WG-6 questions are easy to use, inexpensive and do not require technical expertise; but if information on how reliable they are in actually identifying the intended population is not available, these features are not much of a selling point. Validation of the WG-6 beyond cognitive testing is needed to see if these questions indeed identify the population of interest. At best, the current application of the WG-6 questions makes it possible to determine the frequency in a population of severe problems in six domains of functioning.

Madans, Mont and Loeb cast doubt on the application of Item Response Theory (IRT) methods to create scales of capacity and performance with true metrical properties, in order to directly determine a threshold point of persons who experience severe disability. In part, their doubt is that this statistical exercise requires too high a level of resources and too advanced technical capacity from national statistical offices. Arguing that an acknowledged and broadly used method for developing scales should not be used because of its complexity is questionable. Indeed, we assume that national statistical offices have the background necessary to apply the relatively standard approaches of latent trait analysis. Our manuscript also clearly shows that the assumption behind going for a categorical exercise of counting difficulties, as recommended for the WG-6 screener, namely that people with important difficulties in different functioning domains have the same level of disability, does not hold. Counting difficulties, as proposed for the WG-6 screener, might be, at first glance, a feasible and straightforward method but remains too imprecise and limited to allow for calculating reliable disability estimates for policy and resources allocation decisions. Madans *et al*. stress that the selection of a cut point is critical and might vary for different purposes. We agree that a threshold should be set that is fit for purpose but using an approach that is essentially categorical and has no cardinal properties to select such a threshold means that this approach remains a “counting” exercise.

Madans, Mont and Loeb affirm that “In accordance with the framework proposed by the ICF, the WG-6 was designed to identify a population which, due to difficulty functioning in core domains, is at risk of restricted participation in a non-accommodating environment”. This statement contradicts the International Classification of Functioning, Disability and Health (ICF) [5]. Inferring that difficulties in “core” functioning domains because of a health condition are directly associated with participation restrictions or even the cause of participation restrictions corresponds actually to the “medical model of disability”. While it is true that persons with significant impairments in body functions or significant difficulties in capacity, understood in the ICF as the extent to which health problems affect how people function in multiple domains, might be the most vulnerable and the WG-6 may identify them correctly, this should remain the stated aim. Providing general estimates of prevalence of disability, as defined in the ICF, as well as estimating its impact on people’s lives in terms of restrictions in participation are clearly beyond the scope of the WG-6. In the ICF, the positive or negative impact of a person’s physical, human-built, attitudinal and social environment can make all the difference to a person’s participation, that is, the disability he or she experiences. This understanding is the operating basis for the MDS approach.

Madans, Mont and Loeb also make the point that “monitoring requires measurement at multiple time periods. Cost and burden on data collection systems limits the frequency and amount of information that can be collected… Short sets of questions can be incorporated into these systems so that the information for monitoring is available at multiple points…”. In our understanding, monitoring requires tracking with tools that are sensitive to change over time, again a psychometric property required even from short sets of questions.

There is indeed a cost to data collection and while the WG-6 offer a short set that can be embedded in existing surveys, by identifying a subset of the population at risk for disability it does not mainstream disability and remains an exercise to identify a minority and their potential lack of equal participation when disaggregated for different indicators. This detracts from the fact that at least 15% of the world’s population, over a billion people, are in need of accommodating environments and the right health and social policies—this is not about a very small fraction of the population but a substantial number and hence resources need to be allocated proportionately and the disability agenda needs to be correspondingly mainstreamed.

The WG-6 is an attempt to identify the minority vulnerable population but provides little insights into how to improve their lives. In the long run, this lack of insight can impose much higher costs on the health and social security systems than that needed to run a comprehensive data collection exercise suitable to inform policy and public health interventions.

In the end, any disability data collection technique has to be fit for purpose. If a country’s purpose in collecting data is to determine the frequency of respondents who experience high levels of impairments and decrements in capacity in six basic domains (which may or may not be statistically robust enough to capture this sub-population), then the WG screener questions are a straightforward way to accomplish that.

However, if a country wishes to invest its resources to respond appropriately and adequately to the call of persons with disabilities to enhance their participation and social inclusion through reasonable accommodations, mainstreaming of services and social inclusion, it should direct its resources to collecting robust data in order to inform programmes and policies that maximize the outcome of interest: full participation. These are not merely ideal aspirations but the expressed directives of the United Nations Convention on the Rights of Persons with Disabilities, and targets of several of the Sustainable Development Goals. The MDS strategy, we believe, stands a much better chance of meeting these enormous challenges.
